# Glioblastoma Biology, Genetics and Possible Therapies

**DOI:** 10.3390/cells12162063

**Published:** 2023-08-14

**Authors:** Javier S. Castresana, Bárbara Meléndez

**Affiliations:** 1 Department of Biochemistry and Genetics, University of Navarra School of Sciences, 31008 Pamplona, Spain; 2 Molecular Pathology Research Unit, Virgen de la Salud Hospital, 45005 Toledo, Spain; bmelendez@sescam.jccm.es

Glioblastoma is the most aggressive intracranial tumor. Current treatment consists of surgery, radiotherapy, and chemotherapy (temozolomide). Resistance to radiotherapy and chemotherapy are frequent. Temozolomide is preferably used in patients who do not express MGMT. Temozolomide induces the formation of O6-methylguanine in DNA. MGMT repairs this DNA damage. Therefore, glioblastoma cells become resistant to temozolomide when MGMT expression ensures the repair of damaged DNA by temozolomide. On the contrary, if MGMT is not expressed, the DNA damage caused by temozolomide will not be repaired, and glioblastoma cells will die, that is, glioblastoma cells will be sensitive to temozolomide.

Lack of MGMT expression is associated with the hypermethylation of the MGMT promoter. Therefore, in clinical practice, an immunohistochemical approach is used to detect patients who do not express MGMT in glioblastoma biopsies, being these patients the ones who could benefit the most from MGMT expression laboratory assays [1].

Due to chemotherapy resistance, various combinations of drugs with temozolomide are being tested. The sensitization to temozolomide in previously resistant cells can be seen, thanks to the intervention of a second compound [2].

Bioactive compounds are also tested in order to prove their possible inhibitory activity of cell growth or migration in glioblastoma [3,4,5]. Sun et al. [3] demonstrated the antimetastatic potential of corosolic acid in glioblastoma cells by inhibiting the JAK2/MEK/ERK axis.

Kapoor-Narula and Lenka [4] demonstrated the anticancer effect of Oltipraz, a synthetic dithiolethione present in many vegetables, by decreasing the glioma cancer stem cells content in favor of differentiating GFAP+ glioma cells, together with the suppression of neurospheres formation. Even in vivo treatment with Oltipraz ectopically suppressed glioblastoma tumors xenografted in mice.

Several articles have revealed a preliminary positive effect of cannabinoids against glioblastoma [6,7,8]. Hohmann et al. [5] did not reach the same clear conclusions: on the one hand, they saw that cannabinoids increased the size of glioblastoma spheroids, but on the other hand, migration was unaffected.

Another way of intervention against glioblastoma is the direct attack of its brain tumor stem cells, trusting that such cells are the initiators and/or maintainers of the tumor, as well as being the cells that make the tumor resistant to chemotherapy and radiotherapy. In this sense, there are promising findings, such as those published by Lacore et al. [9], who blocked the M6a glycoprotein by siRNA, leading to a decrease in cell proliferation and invasion, as well as to an increase in radiosensitivity in glioblastoma stem cells.

Also, trying to target the stem cell component of this tumor, Essien et al. [10] assayed a combined treatment of an HDAC and an MEK inhibitor, together with radiation, detecting a bigger decay in the expression of stem cell markers Nestin and SOX2 than with the standard treatment of temozolomide and radiation. Other studies have shown the efficacy of epigenetically inhibiting HDAC in glioblastoma cells [11,12,13,14,15], even preferentially targeting the cancer stem cell compartment [16,17].

Tumor cell dormancy complicates cancer therapy [18]. Cells that have metastasized to other organs remain in a quiescent state even for years, after which the cells might be newly activated and capable of originating the true metastatic disease. Every effort to understand the life cycle of tumor cell dormancy [19,20], the possible similarities found between tumor dormant cells and tumor stem cells [21,22], and, even more important, the sensitization of dormant cells to chemotherapy [23] are of great importance to overcome metastatic disease. Glioblastoma almost never metastasizes outside of the brain. Rather, it invades into the brain, but also develops tumor cell dormancy [20,22,24] capable of producing recurrent tumors several months after complete surgical resection, radiotherapy, and temozolomide treatments. Therefore, research has also been conducted on how glioblastoma dormant cells induced by temozolomide treatment can develop stem cell characteristics: Kubelt et al. [24] reported in this Special Issue about a possible connection between temozolomide-induced glioblastoma cell dormancy and the development of stem-like characteristics in glioblastoma cells.

Several other articles of this Special Issue concentrate on inhibiting specific targets with the idea of inhibiting or reducing cell proliferation, migration, and invasion. Then, Pai et al. [25] revealed that the inhibition of FABP6 (a bile acid carrier protein) reduced invasion and angiogenesis in glioblastoma cells by decreasing MMP-2 and VEGF. Secondly, the expression of XRN2, a 5′-3′ exoribonuclease, was shown to be associated with cell migration and the invasion of glioblastoma cells [26]; therefore, inhibition of XRN2 expression might be a strategy to treat glioblastoma. And thirdly [27], it was revealed that the role of the Warburg effect in cancer cells, which turns on aerobic glycolytic processes and methylglyoxal synthesis, finally provokes a general glycation pattern that leads to the invasion of glioblastoma cells, a mechanism that might be disrupted by deglycating agents.

Another approach to combat glioblastoma might be the possibility of targeting specifically well-known pathways like Sonic Hedgehog, Wnt, Notch, TGFbeta, and others [28]. In such a way, single-cell studies [29], transcriptome analysis [30], and organoid models [31] are good approaches to define a holistic picture of glioblastoma.

Two reviews dealing about the epigenetic role of miRNA [32] and of circular RNA [33] in relation with different pathways that promote glioblastoma lead us into the last known category of epigenetic control, apart from histone methylation, histone acetylation, and DNA gene promoter methylation, all of them playing a role in the genesis of glioblastoma [34,35].

Finally, a new way of leading glioblastoma cells to differentiation is proposed by Hide et al. [36] based on ribosomes and ribosomal protein S6 administered to glioblastoma cells. Those cells might then be differentiated into reprogrammed glioblastoma stem cells with the possibility of the further differentiation of normal cells.

In summary, this second Special Issue on the Molecular and Cellular Mechanisms of Glioblastoma presents 16 articles dealing on the biology, genetics, and possible treatments against this devastating disease. Pathways to gliomagenesis and new targets have been explored, together with epigenetic possibilities like the inhibition of HDAC and the role of miRNA and circular RNA, tumor cell dormancy, cancer stem cells, and other approaches, to try to better understand and possibly combat glioblastoma.

## References

[1] Lalezari S., Chou A.P., Tran A., Solis O.E., Khanlou N., Chen W., Li S., Carrillo J.A., Chowdhury R., Selfridge J. (2013). Combined analysis of O6-methylguanine-DNA methyltransferase protein expression and promoter methylation provides optimized prognostication of glioblastoma outcome. Neuro Oncol..

[2] Tomar M.S., Kumar A., Srivastava C., Shrivastava A. (2021). Elucidating the mechanisms of Temozolomide resistance in gliomas and the strategies to overcome the resistance. Biochim. Biophys. Acta Rev. Cancer.

[3] Sun L.W., Kao S.H., Yang S.F., Jhang S.W., Lin Y.C., Chen C.M., Hsieh Y.H. (2021). Corosolic Acid Attenuates the Invasiveness of Glioblastoma Cells by Promoting CHIP-Mediated AXL Degradation and Inhibiting GAS6/AXL/JAK Axis. Cells.

[4] Kapoor-Narula U., Lenka N. (2022). Elucidating the Anti-Tumorigenic Efficacy of Oltipraz, a Dithiolethione, in Glioblastoma. Cells.

[5] Hohmann U., Walsleben C., Ghadban C., Kirchhoff F., Dehghani F., Hohmann T. (2022). Interaction of Glia Cells with Glioblastoma and Melanoma Cells under the Influence of Phytocannabinoids. Cells.

[6] Rupprecht A., Theisen U., Wendt F., Frank M., Hinz B. (2022). The Combination of Δ(9)-Tetrahydrocannabinol and Cannabidiol Suppresses Mitochondrial Respiration of Human Glioblastoma Cells via Downregulation of Specific Respiratory Chain Proteins. Cancers.

[7] Milian L., Mata M., Alcacer J., Oliver M., Sancho-Tello M., Martín de Llano J.J., Camps C., Galbis J., Carretero J., Carda C. (2020). Cannabinoid receptor expression in non-small cell lung cancer. Effectiveness of tetrahydrocannabinol and cannabidiol inhibiting cell proliferation and epithelial-mesenchymal transition in vitro. PLoS ONE.

[8] Marcu J.P., Christian R.T., Lau D., Zielinski A.J., Horowitz M.P., Lee J., Pakdel A., Allison J., Limbad C., Moore D.H. (2010). Cannabidiol enhances the inhibitory effects of delta9-tetrahydrocannabinol on human glioblastoma cell proliferation and survival. Mol. Cancer Ther..

[9] Lacore M.G., Delmas C., Nicaise Y., Kowalski-Chauvel A., Cohen-Jonathan-Moyal E., Seva C. (2022). The Glycoprotein M6a Is Associated with Invasiveness and Radioresistance of Glioblastoma Stem Cells. Cells.

[10] Essien E.I., Hofer T.P., Atkinson M.J., Anastasov N. (2022). Combining HDAC and MEK Inhibitors with Radiation against Glioblastoma-Derived Spheres. Cells.

[11] De La Rosa J., Urdiciain A., Zazpe I., Zelaya M.V., Meléndez B., Rey J.A., Idoate M.A., Castresana J.S. (2020). The synergistic effect of DZ-NEP, panobinostat and temozolomide reduces clonogenicity and induces apoptosis in glioblastoma cells. Int. J. Oncol..

[12] Chang H.H., Chang Y.Y., Tsai B.C., Chen L.J., Chang A.C., Chuang J.Y., Gean P.W., Hsueh Y.S. (2022). A Selective Histone Deacetylase Inhibitor Induces Autophagy and Cell Death via SCNN1A Downregulation in Glioblastoma Cells. Cancers.

[13] Uddin M.S., Mamun A.A., Alghamdi B.S., Tewari D., Jeandet P., Sarwar M.S., Ashraf G.M. (2022). Epigenetics of glioblastoma multiforme: From molecular mechanisms to therapeutic approaches. Semin. Cancer Biol..

[14] Kunadis E., Lakiotaki E., Korkolopoulou P., Piperi C. (2021). Targeting post-translational histone modifying enzymes in glioblastoma. Pharmacol. Ther..

[15] Chen R., Zhang M., Zhou Y., Guo W., Yi M., Zhang Z., Ding Y., Wang Y. (2020). The application of histone deacetylases inhibitors in glioblastoma. J. Exp. Clin. Cancer Res..

[16] Nakagawa-Saito Y., Saitoh S., Mitobe Y., Sugai A., Togashi K., Suzuki S., Kitanaka C., Okada M. (2022). HDAC Class I Inhibitor Domatinostat Preferentially Targets Glioma Stem Cells over Their Differentiated Progeny. Int. J. Mol. Sci..

[17] Reddy R.G., Bhat U.A., Chakravarty S., Kumar A. (2020). Advances in histone deacetylase inhibitors in targeting glioblastoma stem cells. Cancer Chemother. Pharmacol..

[18] Gomis R.R., Gawrzak S. (2017). Tumor cell dormancy. Mol. Oncol..

[19] Phan T.G., Croucher P.I. (2020). The dormant cancer cell life cycle. Nat. Rev. Cancer.

[20] Adamski V., Hattermann K., Kubelt C., Cohrs G., Lucius R., Synowitz M., Sebens S., Held-Feindt J. (2020). Entry and exit of chemotherapeutically-promoted cellular dormancy in glioblastoma cells is differentially affected by the chemokines CXCL12, CXCL16, and CX3CL1. Oncogene.

[21] Hen O., Barkan D. (2020). Dormant disseminated tumor cells and cancer stem/progenitor-like cells: Similarities and opportunities. Semin. Cancer Biol..

[22] Adamski V., Hempelmann A., Flüh C., Lucius R., Synowitz M., Hattermann K., Held-Feindt J. (2017). Dormant glioblastoma cells acquire stem cell characteristics and are differentially affected by Temozolomide and AT101 treatment. Oncotarget.

[23] Carlson P., Dasgupta A., Grzelak C.A., Kim J., Barrett A., Coleman I.M., Shor R.E., Goddard E.T., Dai J., Schweitzer E.M. (2019). Targeting the perivascular niche sensitizes disseminated tumour cells to chemotherapy. Nat. Cell Biol..

[24] Kubelt C., Hellmold D., Esser D., Ahmeti H., Synowitz M., Held-Feindt J. (2023). Insights into Gene Regulation under Temozolomide-Promoted Cellular Dormancy and Its Connection to Stemness in Human Glioblastoma. Cells.

[25] Pai F.C., Huang H.W., Tsai Y.L., Tsai W.C., Cheng Y.C., Chang H.H., Chen Y. (2021). Inhibition of FABP6 Reduces Tumor Cell Invasion and Angiogenesis through the Decrease in MMP-2 and VEGF in Human Glioblastoma Cells. Cells.

[26] Dang T.T., Lerner M., Saunders D., Smith N., Gulej R., Zalles M., Towner R.A., Morales J.C. (2022). XRN2 Is Required for Cell Motility and Invasion in Glioblastomas. Cells.

[27] Schildhauer P., Selke P., Scheller C., Strauss C., Horstkorte R., Leisz S., Scheer M. (2023). Glycation Leads to Increased Invasion of Glioblastoma Cells. Cells.

[28] Drakulic D., Schwirtlich M., Petrovic I., Mojsin M., Milivojevic M., Kovacevic-Grujicic N., Stevanovic M. (2022). Current Opportunities for Targeting Dysregulated Neurodevelopmental Signaling Pathways in Glioblastoma. Cells.

[29] Lessi F., Franceschi S., Morelli M., Menicagli M., Pasqualetti F., Santonocito O., Gambacciani C., Pieri F., Aquila F., Aretini P. (2022). Single-Cell Molecular Characterization to Partition the Human Glioblastoma Tumor Microenvironment Genetic Background. Cells.

[30] Lozinski M., Bowden N.A., Graves M.C., Fay M., Day B.W., Stringer B.W., Tooney P.A. (2022). Transcriptomic Profiling of DNA Damage Response in Patient-Derived Glioblastoma Cells before and after Radiation and Temozolomide Treatment. Cells.

[31] Weth F.R., Peng L., Paterson E., Tan S.T., Gray C. (2022). Utility of the Cerebral Organoid Glioma ‘GLICO’ Model for Screening Applications. Cells.

[32] Hasan H., Afzal M., Castresana J.S., Shahi M.H. (2023). A Comprehensive Review of miRNAs and Their Epigenetic Effects in Glioblastoma. Cells.

[33] Ahmed S.P., Castresana J.S., Shahi M.H. (2022). Role of Circular RNA in Brain Tumor Development. Cells.

[34] Montella L., Cuomo M., Del Gaudio N., Buonaiuto M., Costabile D., Visconti R., Di Risi T., Vinciguerra R., Trio F., Ferraro S. (2023). Epigenetic alterations in glioblastomas: Diagnostic, prognostic and therapeutic relevance. Int. J. Cancer.

[35] Romani M., Pistillo M.P., Banelli B. (2018). Epigenetic Targeting of Glioblastoma. Front. Oncol..

[36] Hide T., Shibahara I., Inukai M., Shigeeda R., Kumabe T. (2022). Ribosomes and Ribosomal Proteins Promote Plasticity and Stemness Induction in Glioma Cells via Reprogramming. Cells.

